# Supplementary material for: Deciphering the Dual Role of Heligmosomoides polygyrus Antigens in Macrophage Modulation and Breast Cancer Cell Growth
Source: Vet Sci. 2024 Feb 3;11(2):69. doi: 10.3390/vetsci11020069 (PMC10891978; doi:10.3390/vetsci11020069)

Supplementary Table S1. Sequences of starters used to perform qPCR.

Gene	Primerbank ID	Forward Primer	Reverse Primer	Amplicon Size [bp]
Arg1	7106255a1	CTCCAAGCCAAAGTCCTTAGAG	AGGAGCTGTCATTAGGGACATC	185
NOS2	6754872a1	GTTCTCAGCCCAACAATACAAGA	GTGGACGGGTCGATGTCAC	127
IL-4	10946584a1	GGTCTCAACCCCCAGCTAGT	GCCGATGATCTCTCTCAAGTGAT	102
Chil3 (YM1)	6753416a1	CAGGTCTGGCAATTCTTCTGAA	GTCTTGCTCATGTGTGTAAGTGA	197
IL-10	6754318a1	GCTCTTACTGACTGGCATGAG	CGCAGCTCTAGGAGCATGTG	105
IL-6	13624311a1	TAGTCCTTCCTACCCCAATTTCC	TTGGTCCTTAGCCACTCCTTC	76
TNF-alfa	7305585a1	CCCTCACACTCAGATCATCTTCT	GCTACGACGTGGGCTACAG	61
Mrc1 (CD206)	6678932a1	CTCTGTTCAGCTATTGGACGC	CGGAATTTCTGGGATTCAGCTTC	132
CCL2	6755430a1	TTAAAAACCTGGATCGGAACCAA	GCATTAGCTTCAGATTTACGGGT	121
PPIA	6679439a1	GAGCTGTTTGCAGACAAAGTTC	CCCTGGCACATGAATCCTGG	125

Supplementary Figure S1

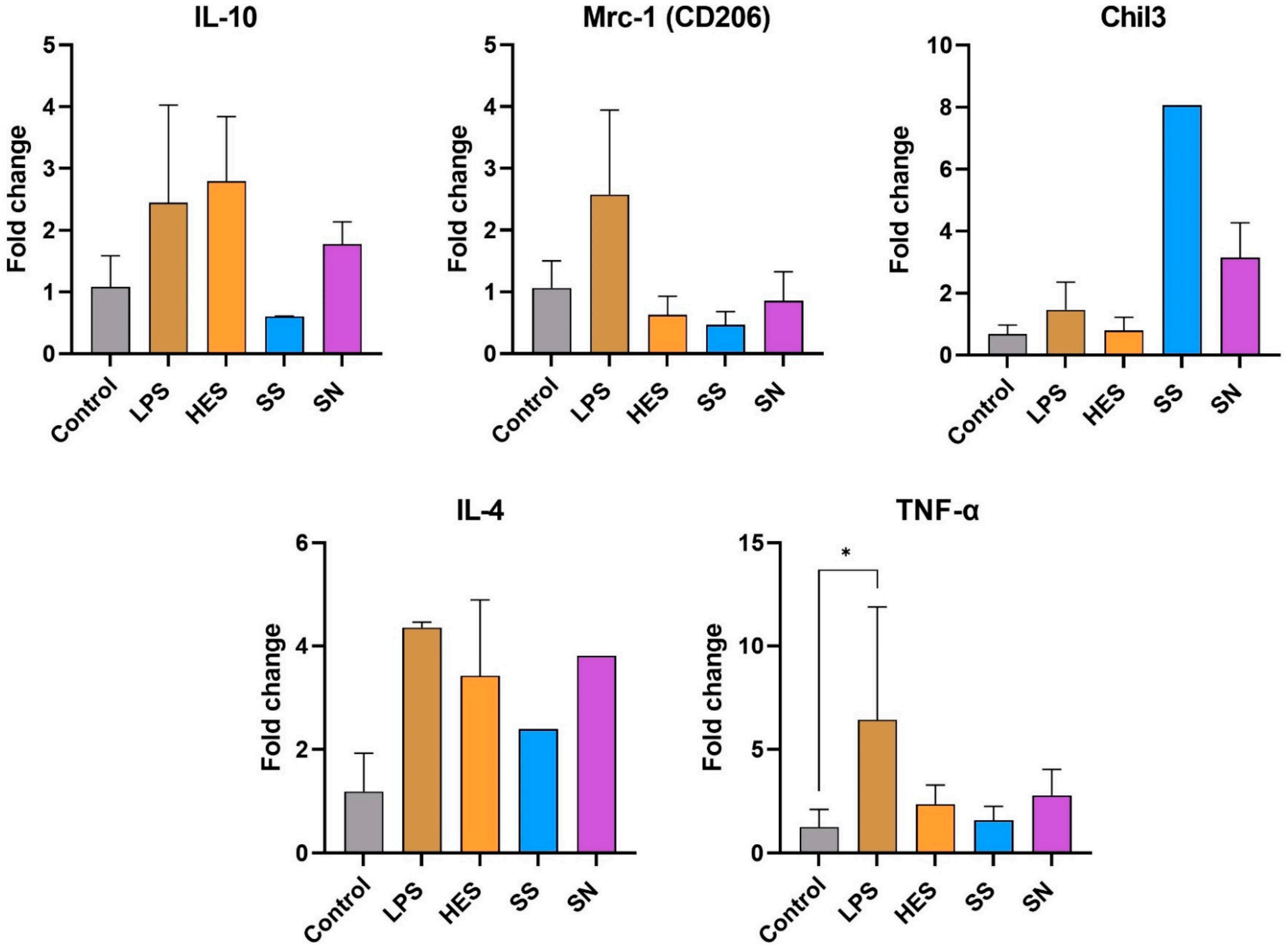

Supplement: Supplementary file 1 [file vetsci-11-00069-s001.zip › vetsci-2773644-supplementary.pdf]
